# A Digital Decision Support Tool to Enhance Decisional Capacity for Clinical Trial Consent: Design and Development

**DOI:** 10.2196/10525

**Published:** 2018-06-06

**Authors:** Robert D Furberg, Alexa M Ortiz, Rebecca R Moultrie, Melissa Raspa, Anne C Wheeler, Lauren A McCormack, Donald B Bailey Jr

**Affiliations:** ^1^ Digital Health and Clinical Informatics RTI International Research Triangle Park, NC United States; ^2^ Patient and Family Engagement RTI International Research Triangle Park, NC United States; ^3^ Center for Newborn Screening, Ethics, and Disability Studies RTI International Research Triangle Park, NC United States; ^4^ Public Health Research Division RTI International Research Triangle Park, NC United States

**Keywords:** decision support, informed consent, digital health, intellectual disability, fragile X syndrome, telemedicine

## Abstract

**Background:**

Challenges in the clinical and research consent process indicate the need to develop tailored, supportive interventions for all individuals, especially those with limited decisional capacity. We developed a tool to enhance shared decision making and the decisional capacity for individuals with fragile X syndrome engaged in the informed consent process for a clinical trial.

**Objective:**

We describe the design and development process of a tablet-based decision support tool.

**Methods:**

Our development process for the decision support tool employed a user-centered, feature-driven design approach. We began with an environmental scan to catalog relevant mobile apps, and we conducted interviews with people with a diagnosis of fragile X syndrome and clinicians at fragile X syndrome clinics. To develop content for the decision support tool, we extracted key concepts and elements from a real clinical trial consent form and rewrote it using plain-language principles.

**Results:**

We used iterative testing to continuously evaluate and revise the decision support tool content. The tool was finalized in 2016 and contained a series of vignettes, quiz questions, and a sorting activity. A randomized controlled trial was then conducted to compare the efficacy of the decision support tool with a standard verbal presentation of material that mimicked typical informed consent practice.

**Conclusions:**

The informed consent process is primed to leverage digital health resources that promote increased understanding and engagement of research participants in the consent and research process. The process and experiences we describe may provide a model for other digital health design and development initiatives seeking to create more interactive and accessible decision support resources.

**Trial Registration:**

ClinicalTrials.gov NCT02465931; https://www.clinicaltrials.gov/ct2/show/NCT02465931 (Archived by WebCite at http://www.webcitation.org/6zx2KY9YW)

## Introduction

### Digital Health and Decision Support

Digital technologies can serve as a communication bridge between patients, caregivers, and health care providers, making information available to users when and where they need it, and allowing users to better communicate their needs and preferences. For those with intellectual and developmental disabilities, technological advances can be used to support daily living skills, enhance cognition, and support communication. Further, multimedia formats for information delivery—including interactive, computer-based interventions—may contribute to greater patient understanding of complex information when compared with traditional formats [1-4] and are gaining in popularity [5].

Electronic informed consent strategies use electronic media (such as websites, video, or audio) to convey study information and obtain the participant’s consent [5]. Researchers are beginning to see the potential value of electronic informed consent methods as opposed to traditional paper-based methods.

Prospective research participants sometimes struggle to comprehend informed consent standards and regulations [6]. One such challenge is a lack of general understanding of the research and important concepts [1,7]. Participants also struggle to understand the potential risks and benefits of research [7], and to understand their rights, the treatment they may receive [7], and the purpose of the research for which they are being asked to provide their consent [8,9]. Informed consent documents and informational materials for patients focus more on meeting minimal ethical requirements than facilitating the decision-making process [10]. Audiovisual interventions may have the potential to provide benefits to the informed consent process by improving participant understanding and satisfaction [11].

### Overview of Fragile X Syndrome

Fragile X syndrome (FXS) is the leading inherited type of intellectual disability. Males with a diagnosis of FXS typically have impairment ranging from mild to severe; females are generally less impaired [12]. This wide range of cognitive skills among those with FXS can result in variable decisional capacity and the ability to make choices [13].

To date, most research on individuals with FXS has been noninvasive, limited to parent surveys and secondary assessment of clinical data [13,14]. Studies such as these typically involve straightforward consent or assent processes or parental consent. However, with advances in understanding the underlying science of FXS, the number of clinical trials available for individuals with FXS has increased [13,15]. Decisions related to enrollment in treatment trials are now more complex than in the past; thus, researchers are compelled to consider how best to support decision making for individuals who present with a range of decisional capacity. Recent technological advances in digital health have the potential to dramatically change the consenting process for those with FXS.

### Decision Making and Fragile X Syndrome

The knowledge base surrounding the decisional capacity of those with intellectual disability and FXS is inadequate, and reviews concluded that the literature is limited in both scope and focus [14,16]. The few studies that examined ways to support individuals with intellectual disability in the informed consent process found that the presentation of information is important, given that language skills, memory, and previous decision making all have an impact on the ability to consent [16]. Due to the wide range in decisional capacity, those with intellectual disability can participate in the consent process, but many authors encourage that participation should be determined and supported on an individual case-by-case basis [13,16,17]. The use of digital decision support tools can potentially improve the understanding of clinical trial consent for those with FXS. The purpose of this paper is to describe the design and development process of a tablet-based decision support tool to enhance shared decision making and decisional capacity for those with FXS participating in the informed consent process.

## Methods

### Design Process

**Figure 1:**
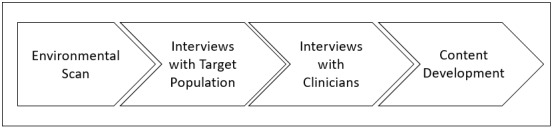
Methodology for decision support tool content development.

The user-centered design process outlines the design and development life cycle focused on gaining a deep understanding of a system’s end users. A variety of user-centered design guidelines are available to inform the development of digital technologies. For example, the international standard 9241-210:2010 [18] provides the requirements and recommendations for human-centered design principles to guide the development of computer-based interactive systems. Several US federal resources to support implementation and management of user-centered design are freely available from the United States Digital Service [19], 18F [20], and usability.gov [21]. Our team leveraged these resources to develop a tablet-based decision support tool. Figure 1 outlines the methodology our project team used to identify and develop content for the tool.

### Environmental Scan

To begin, our team conducted an environmental scan to catalog available tablet-based apps that focus on health care or were designed for individuals with intellectual and developmental disabilities. Our goal was to evaluate the apps based on user-centered design principles and determine what features we needed to include when developing the tool. Study staff purchased an iPad (Apple Inc, Cupertino, CA, USA) and identified 31 apps (see Multimedia Appendix 1) categorized as follows: (1) communication apps (n=10), (2) educational and social skills apps (n=8), (3) decision support apps (n=7), (4) clinical trial apps (n=3), and (5) behavior modification apps (n=3). Based on our review, we formulated recommendations for key features, outlined in Table 1. Although the recommendations do not encompass all considerations necessary for tool development, they provide a well-rounded initial assessment of features either that are currently used by or with individuals of our target population, or that need to be developed and enhanced to address inequities for a successful informed consent decision support tool.

**Table 1:** Recommended key features of apps.

Decision support tool feature	Feature description
Apply clear communication and plain-language principles	The tool should reflect clear communication principles (eg, avoid jargon, use a low reading level) and be easy to understand.
Ensure appropriateness to sensitivities	Content and presentation elements need to be respectful of particular sensitivities common among individuals with fragile X syndrome (eg, heightened sensitivity to light, color, and sound).
Combine animation and real-life images	The combination of animation and real-life images provides engagement while grounding concepts in the real world and provides tangible orientation to relevant scenarios (eg, a clinic waiting room).
Enable customization	Customization of the content and delivery should be enabled to ensure accessibility to a broader audience.
Incorporate active learning	Active learning principles should be incorporated to facilitate greater engagement and integration of the information.
Assess comprehension	Existing methods (eg, the “teach back” method) should be incorporated or new ways should be developed to assess a user’s comprehension of information received to gauge the effectiveness of the tool.
Support decision making	Simple decision support tools should be offered to facilitate reasoning about a decision (eg, a pro/con list) and assessment of preference (eg, importance of factors) related to that decision.

### Interviews With the Target Population

In the second step to inform the appropriate features and functionality for the decision support tool, we conducted 6 in-person observation-based interviews with individuals with a diagnosis of FXS. This was a convenience sample of participants identified through a larger study on health care decision making among individual with FXS; 5 participants were male and their average age was 22.3 years (range 16-28 years). Participants were given an iPad and rated on their engagement and performance of simple skills, advanced skills, and exploration skills interacting with specific apps. Overall, all participants interacted with the assessment apps and were most engaged with exploring app hotspots that involved avatars or narration, and least engaged with simplistic app features. Results from these interviews will be published at a later date.

### Interviews With Clinicians

To establish a better understanding of the context within which the decision support tool would be used, we conducted 3 in-depth interviews with clinician stakeholders who had taken participants with FXS through the consent process for clinical trials. This was a convenience sample of clinicians or physician scientists who were known members of our project team and willing to serve as consultants, and to provide feedback on the content creation, as well as the design and development efforts throughout the life cycle of the project. From the interviews, it was unclear whether there is a standard or maximum reading level for consent forms. One FXS clinical trial research manager stated that their consent forms were written for an eighth-grade reading level, and the other 2 clinicians noted that their forms include simple questions (possibly at a second- or third-grade reading level) to prompt a yes-or-no response from the patient. It was also the consensus that most individuals with FXS don’t understand much of the information presented to them; however, they are able to understand that they will be taking a new medication, and they are able to understand the risks and benefits of that new medication. Although none of the clinicians regularly used tablets as part of the informed consent process, one clinician emphasized that keeping the participant happy and engaged is the greatest challenge, and they welcomed anything to make the process easier.

**Table 2:** Sample decision support tool content mapping.

Institutional review board element	Real clinical trial consent	Hypothetical clinical trial consent	Decision support tool content
Description of risks or discomforts to subject.	Risks are possible side (adverse) effects from the study drug, other drugs, taking the blood pressure or taking blood.	The new medication is generally considered to be very safe, but one purpose of the study is to determine whether any serious side effects occur. The most common side effects expected are fatigue and a mild headache.	You might not like some parts of the study. If you get the real pills, you might feel a little sick or tired. You also might not like getting your blood drawn.
Description of voluntary compensation and treatment if the subject is injured related to the research. Applicable for research posing greater than minimal risks.	Each study subject will receive US $200 per study visit when you have to stay overnight and US $120 for other visits to the study center, to compensate for your time.	US $25 will be given for each study visit and US $10 for each phone call.	You will get US $25 after each visit. Your name and information about you will be kept private.

### Content Development

Content development began with a review of informed consent forms from previously conducted FXS clinical trials. Although the tool focused on a hypothetical trial, we used the actual consent forms as a guide to extract key concepts and elements that would also be needed in our tool (eg, randomization, blinding, and use of a placebo, as well as concepts that anecdotally are difficult to comprehend and typically explained with medical terminology and jargon). We rewrote these concepts and elements using plain-language principles and incorporating other recommendations we identified in the environmental scan. We used a table to map institutional review board requirements and the clinical trial consent content with language in the decision support tool to ensure that we addressed all mandatory elements of informed consent disclosure. Table 2 shows a sample of how these elements were mapped. We consulted members of the institutional review board of RTI International, Research Triangle Park, NC, USA, to validate this process.

To aid in the development of closed-ended quiz questions for the tool, we adapted the MacArthur Competence Assessment Tool for Clinical Research, which is the main measure of decisional capacity in individuals with FXS [22]. Finally, we developed a sorting activity to identify the perceived reasons (both positive and negative) an individual may consent to participate in a clinical trial.

As the next step in our development process, we created audiovisual components to accompany the content of the tool. Universal design can be defined as a tool that is accessible and usable by everyone [23]. The approach stresses user awareness and emphasizes designs that can be used by as many people as possible while minimizing the need to adapt the product to support particular users, especially those with disabilities or limited function [23]. To develop the imagery and interaction model for the decision support tool, a graphic design artist created draft storyboards of initial content, audio, and a user interface that adhered to the principles of universal design. We sought feedback on the storyboard from the project consultants, stakeholders, individuals with FXS, and their family members on the draft content. We undertook a collaborative and iterative process of refining and ultimately finalizing the content for the decision support tool.

## Results

**Figure 2:**
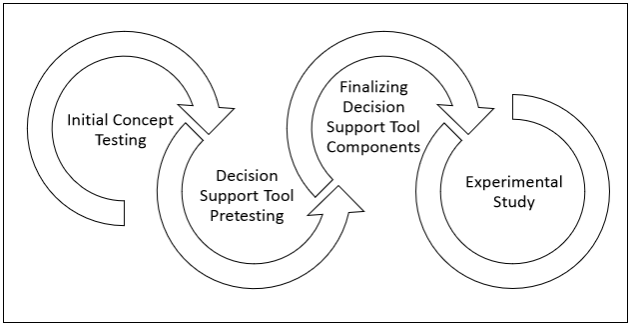
Decision support tool testing.

The results section focuses on initial testing of the decision support tool and how feedback received throughout each phase of testing further influenced the content and design of the tool. Figure 2 outlines our project team’s iterative testing and refinement approach that we used to enhance the decision support tool beyond what we had developed using the user-centered design process described in the Methods section.

### Initial Concept Testing

**Figure 3:**
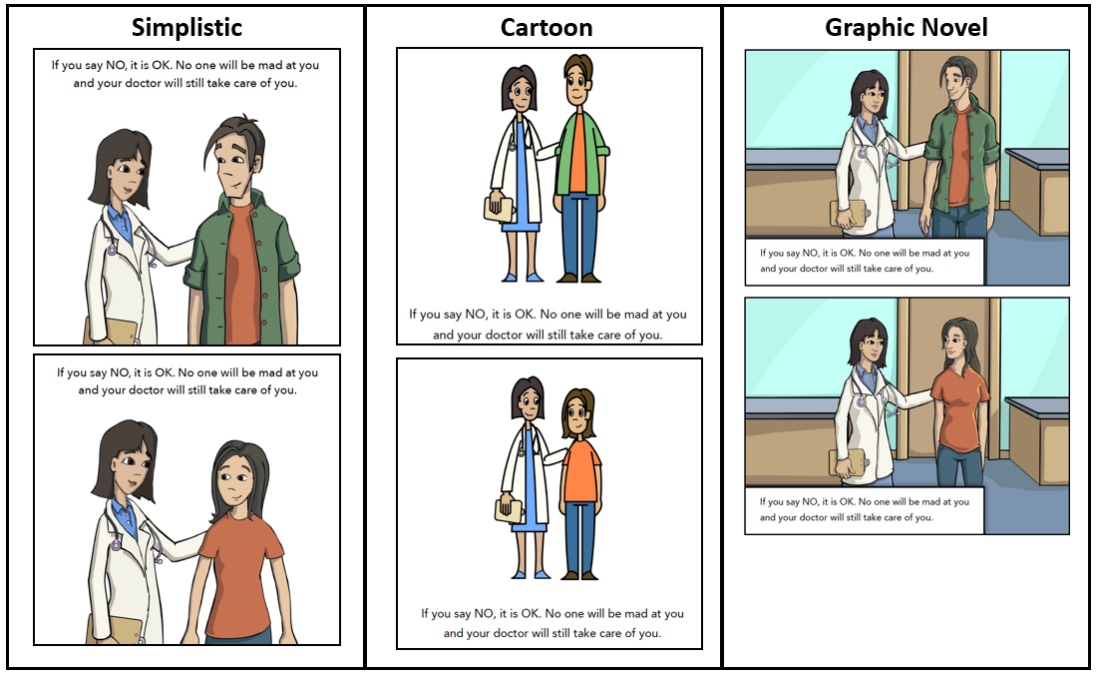
Decision support tool graphic styles.

We sought input from individuals with FXS on the 3 stylistic options for the decision support tool. We displayed a sample of each graphic style (simplistic, cartoon, and graphic novel, as Figure 3 shows) and asked participants to vote on which style they most preferred. A total of 104 participants provided input on their preferred graphic style. Most, 45.2% (n=47), preferred the cartoon style, 36.5% (n=38) preferred the simplistic style, and the remaining 18.3% (n=19) preferred the graphic novel style.

Study staff also conducted in-depth, in-person interviews with 9 individuals with FXS to seek feedback on an early iteration of the tool’s content. Interviews focused mainly on learning whether the images, text, and narration captured the clinical trial component as intended. Interviewers also asked the participants their opinions about the graphics used, suggestions for improvement, and whether the text and narration were understandable. Lower-functioning males with FXS expressed a preference for the cartoon graphic style; however, higher-functioning participants preferred the simplistic style, and we ultimately selected that design in order to appeal to these users.

Our interviews also revealed scenes that required modification and enhancements to increase comprehension among individuals with FXS. For one particular scene, we tested participants’ comprehension of the clinical trial concept of placebo with an animation showing that some pills will contain medicine and others will not. Although participants liked the animation, they had difficulty grasping the concept. Participants also had difficulty understanding the concept of blinding, and that no one will know who will receive the trial drug. Similarly, participants were also confused by the concept of randomization, particularly regarding who decides which trial participant receives the drug versus the placebo. Participants had an easier time understanding more concrete concepts such as trial procedures (eg, providing a urine sample or having blood drawn), and they were able to easily navigate through the different screens on the iPad and liked the narration and animations included throughout the tool.

### Decision Support Tool Content

In the fall of 2015, we completed a draft of the tool composed of a series of 6 vignettes or interactive narratives, close-ended multiple-choice quiz questions, and a sorting activity. Each vignette discussed a separate component of the consent using plain language: study purpose, study involvement, how the study will work, study benefits, study risks, and withdrawing from the study. To evaluate each user’s understanding of the content, multiple-choice questions followed each vignette. Before answering the multiple-choice questions, users were given the option to watch the vignette again. If they answered the questions incorrectly, the vignette automatically replayed for the user 1 time and the multiple-choice question was presented again. A sorting activity was also used to facilitate a self-directed values clarification of the perceived reasons an individual may choose to participate or not participate in a clinical trial. Users were provided with 7 features of study participation (eg, “I would have to see my doctor several times” or “I might feel better”) and asked to sort each feature as a reason to be or not be in the study. Participants were required to sort a minimum of 2 features.

### Pretesting and Finalizing the Decision Support Tool

We conducted incremental field testing on each component of the draft decision support tool: the vignettes, the quiz questions, and the sorting activity. On completion of the initial series of vignettes and in parallel with development of the quiz items, we pretested each component of the tool. We collected feedback from pretesting in a subsequent version while fielding the quiz component; we implemented input on the quiz during development of the sorting activity until we assembled the final decision support tool. The complete decision support tool underwent beta testing and internal software quality assurance testing to exercise the compiled decision support tool, verify skip logic, and confirm capture of accurate scoring metrics and session analytics. We completed the final version of the decision support tool in 2016.

### Experimental Study

We initiated a two-arm randomized controlled trial (NCT02465931) in 2016 to compare the efficacy of the decision support tool with a standard verbal presentation of the consent material that mimicked typical consent practice. Participants were randomly assigned to receive the tablet-based decision support tool or the verbal script and paper consent. The trial protocol and outcomes will be published at a later date.

## Discussion

### Health Technologies to Support Complex Decision Making

The movement to empower patients through health technology to support complex decision making is gaining momentum. As the number of clinical trials targeting those with FXS increases, the goal of involving participants in the decision-making process will become increasingly important, emphasizing the need for tools that allow trial participants to become active members in the decision-making process. The process we describe strives to encourage those conducting trials with FXS patients to reevaluate how their participants are involved in the consenting process. The methods we employed in the design and development of the decision support tool described here can be developed, tested, and incorporated into routine practice. Further, although those with intellectual disability face challenges in making health decisions, those without such impairments are not immune to similar struggles.

Health literacy is defined as an individual’s ability to obtain, process, and understand health information and to use it to make health-related decisions [24]. Low health literacy has been shown to be a systemic issue in the general population. The National Assessment of Adult Literacy [25] found that only 12% of US adults had proficient health literacy. This evidence illuminates deficits among most individuals who are seeking care from health care providers and are considering participation in clinical trials. Our decision support tool speaks to the potential benefits an interactive tool can provide for those making trial participation decisions, regardless of cognitive ability.

### Digital Tools in the Informed Consent Process

The informed consent process is primed to leverage digital health resources given recent changes to the Common Rule in the United States that promote increasing understanding and engagement of research participants in the consent and research process. Interactive electronic informed consent material provides more adaptable content than traditional paper-based materials. The digital decision support tool can be deployed in a variety of settings, such as inpatient and outpatient clinics, hospitals, research facilities, or at home. The home setting enables a prospective trial participant to learn about the trial in a familiar and comfortable setting without perceiving potential undue pressure from medical or research personnel. The ability to go through the consent process at home also fosters shared decision making, as family members or those important to the individual can more easily review and openly discuss the information together. Additionally, the ability to use the tool at home provides convenience and reduces the need for travel to a clinic or physician’s office, which may be difficult for some individuals due to their living situation, financial status, or health issues, or the trial location.

### Use of Agile Development for National Institutes of Health–Sponsored Studies

Agile software development is a group of methods in which requirements and solutions evolve through collaboration between self-organizing, cross-functional teams [26]. It promotes adaptive planning, evolutionary development, early delivery, and continuous improvement, enabling rapid and flexible response to change. Feature-driven development is an iterative and incremental software development process [27]. It is a lightweight, agile method for developing software that blends several industry-recognized best practices into a cohesive whole. These practices are driven from a client-valued functionality (feature) perspective to deliver tangible, working software repeatedly and in a timely manner.

Our development process for the decision support tool was consistent with an agile, feature-driven process. This can deliver value and yield a more efficient, responsive product, all while conforming to mandatory research processes such as evidence reviews, stakeholder engagement, regulatory compliance, and protection of human participants.

### Involvement of an Interdisciplinary Team

The principle of “team science” addresses barriers associated with intervention development and implementation through engagement of an interdisciplinary team. This tactic brings together a variety of researchers with specialized expertise, approaches, and methodologies to solve complex problems [28,29]. The effectiveness of team science is evident in the evolution of multiuniversity research teams, which often produce higher-impact research than do individual investigators [30]. Our project used a team science approach to sustain members’ involvement and inform each phase of development for the decision support tool.

A team science approach is especially critical when considering digital health interventions, which require input and coordination from information technologists, researchers, and health care professionals [28,29]. In line with the team science approach, the development and implementation of the tablet-based decision support tool integrated input from diverse sources. Contributors consisted of clinicians, clinical implementation specialists, communication scientists, regulatory compliance experts, graphic designers, programmers, and field interview staff. We approached development of the tool as an integrated team and remained integrated through completion of the randomized controlled trial.

### Conclusion

Central to the success of this project were the team’s recognition of the importance of a user-centered approach, stakeholder engagement and input, appreciation of interdisciplinarity, and willingness to explore and adapt commercial software methods and management techniques. The process and experiences described here may provide a model for other digital health design and development initiatives seeking to create more interactive and accessible decision support resources. Future research is needed on the impact of decision support tools in obtaining electronic informed consent and their influence on shared decision making and the user’s decisional capacity.

## Appendix

Multimedia Appendix 1 Mobile app environmental scan results.

## Abbreviations

FXS: fragile X syndrome

## References

[1] Tait AR, Voepel-Lewis T, Levine R (2015). Using digital multimedia to improve parents' and children's understanding of clinical trials. Arch Dis Child.

[2] Hermann M (2002). [3-dimensional computer animation--a new medium for supporting patient education before surgery. Acceptance and assessment of patients based on a prospective randomized study--picture versus text]. Surgeon.

[3] Tait AR, Voepel-Lewis T, McGonegal M, Levine R (2012). Evaluation of a prototype interactive consent program for pediatric clinical trials: a pilot study. J Am Med Inform Assoc.

[4] Tait AR, Voepel-Lewis T, Moscucci M, Brennan-Martinez CM, Levine R (2009). Patient comprehension of an interactive, computer-based information program for cardiac catheterization: a comparison with standard information. Arch Intern Med.

[5] Food and Drug Administration (2016). Use of electronic informed consent. Questions and answers: guidance for institutional review boards, investigators, and sponsors.

[6] Grady C, Cummings SR, Rowbotham MC, McConnell MV, Ashley EA, Kang G (2017). Informed consent. N Engl J Med.

[7] Barrett R (2005). Quality of informed consent: measuring understanding among participants in oncology clinical trials. Oncol Nurs Forum.

[8] Williams BF, French JK, White HD, HERO-2 consent substudy investigators (2003). Informed consent during the clinical emergency of acute myocardial infarction (HERO-2 consent substudy): a prospective observational study. Lancet.

[9] Joffe S, Cook EF, Cleary PD, Clark JW, Weeks JC (2001). Quality of informed consent in cancer clinical trials: a cross-sectional survey. Lancet.

[10] Armstrong N, Dixon-Woods M, Thomas A, Rusk G, Tarrant C (2012). Do informed consent documents for cancer trials do what they should? A study of manifest and latent functions. Sociol Health Illn.

[11] Synnot A, Ryan R, Prictor M, Fetherstonhaugh D, Parker B (2014). Audio-visual presentation of information for informed consent for participation in clinical trials. Cochrane Database Syst Rev.

[12] Raspa M, Wheeler AC, Riley C (2017). Public health literature review of fragile X syndrome. Pediatrics.

[13] Bailey DB Jr, Raspa M, Wheeler A, Edwards A, Bishop E, Bann C, Borasky D, Appelbaum PS (2014). Parent ratings of ability to consent for clinical trials in fragile X syndrome. J Empir Res Hum Res Ethics.

[14] Cleaver S, Ouellette-Kuntz H, Sakar A (2010). Participation in intellectual disability research: a review of 20 years of studies. J Intellect Disabil Res.

[15] Ligsay A, Hagerman R, Berry-Kravis E, Willemsen R, Kooy RF (2017). Overview of targeted double-blind, placebo-controlled clinical trials in fragile X syndrome. Fragile X Syndrome: From Genetics to Targeted Treatment. First edition.

[16] Goldsmith L, Skirton H, Webb C (2008). Informed consent to healthcare interventions in people with learning disabilities--an integrative review. J Adv Nurs.

[17] Veenstra MY, Walsh PN, van Schrojenstein Lantman-de Valk HM, Haveman MJ, Linehan C, Kerr MP, Weber G, Salvador-Carulla L, Carmen-Cara A, Azema B, Buono S, Germanavicius A, Tossebro J, Maatta T, van Hove G, Moravec D (2010). Sampling and ethical issues in a multicenter study on health of people with intellectual disabilities. J Clin Epidemiol.

[18] International Organization for Standardization (2010). Ergonomics of human-system interaction -- Part 210: Human-centred design for interactive systems (ISO 9241-210:2010).

[19] U.S. Digital Service Building a more awesome government through technology.

[20] 18F (2018). 18F partners with federal agencies to improve the user experience of government.

[21] (2018). usability.gov.

[22] Appelbaum PS, Grisso T (2001). MacArthur Competence Assessment Tool for Clinical Research.

[23] GSA Government-wide Section 508 Accessibility Program.

[24] National Network of Libraries of Medicine Health literacy.

[25] Kutner M, Greenberg E, Jin Y, Paulsen C (2006). The health literacy of America’s adults: results from the 2003 National Assessment of Adult Literacy (NCES 2006–483). U.S. Department of Education.

[26] Shore J (2007). The Art of Agile Development: Pragmatic Guide to Agile Software Development.

[27] Palmer SR, Felsing M (2001). A Practical Guide to Feature-Driven Development.

[28] Burrus O, Gupta C, Ortiz A, Harshbarger C (2018). The Medical Care Blog.

[29] Burrus O, Gupta C, Ortiz A, Zulkiewicz B, Furberg R, Uhrig J, Harshbarger C, Lewis M (2018). Principles for developing innovative HIV digital health interventions: the case of Positive Health Check. Med Care.

[30] Jones BF, Wuchty S, Uzzi B (2008). Multi-university research teams: shifting impact, geography, and stratification in science. Science.

