# Supplementary material for: A Digital Decision Support Tool to Enhance Decisional Capacity for Clinical Trial Consent: Design and Development
Source: JMIR Res Protoc. 2018 Jun 6;7(6):e10525. doi: 10.2196/10525 (PMC6010840; doi:10.2196/10525)
Supplement: Multimedia Appendix 1 [file resprot_v7i6e10525_app1.pdf]

Category	Mobile Application	Description
Communication (n=10)	AutisMate	This app allows users to take their own videos, pictures, and voice recordings to share with others on the Autism spectrum.
	AutismXpress	This app uses colorful, animated icons which can be used by those with Autism to help express their emotions.
	First Then Visual Schedule	This app is used for people who require a structured environment and assists with activity transitions by providing a visual schedule.
	Grid Player	This app allows users to build sentences by selecting a specific symbol or button. These sentences are then vocalized by the app.
	Look2Learn - AAC	Individuals use photographs in this app to express their needs and wants. The app also comes with pre-loaded vocal outputs that users can pair with photographs.
	Pocket Cancer Care Guide	This app was designed for cancer patients or caregivers of cancer patients to easily compile questions for their physician.
	Proloquo	This app is a Multilingual Augmentative and Alternative Communication (AAC) solution designed for those who are unable to speak or have difficulty communicating. This software was designed for Mac OS X.
	Proloquo2Go	This app allows users to build sentences by selecting a specific symbol or button. These sentences are then vocalized by the app.
	Sono Flex Lite	This app allows users to build sentences by selecting a specific symbol or button. These sentences are then vocalized by the app.
	TalkingTILES	This app allows users to build sentences by selecting a specific symbol or button. These sentences are then vocalized by the app.
Educational/ Social Skills (n=8)	Autism Apps Behavior Scripts	This app describes a variety of social situations children with Autism may encounter. The app also provides behavioral options for children to choose from.
	Conversation Social Stories and	This app teaches communication skills

	Simple PECS Comm Tool	through four social stories about different conversational skills.
	Hidden Curriculum for Kids	This app includes entries about “unwritten social rules” that may result in anxiety or confusion for those with Autism.
	Living Safely	This app provides self-directed learning sessions on home and personal safety skills topics.
	Luca Lashes Visits the Doctor	This app helps children learn what to expect from a visit to the doctor through interactivity, touch, sight, and sound.
	My Health, My Choice, My Responsibility	This is a cognitively accessible self-directed learning app that helps individuals with special needs learn about eight topics necessary for maintaining a healthy lifestyle.
	Stories2Learn	This app uses stories to help promote social messages to individuals with autism and other developmental disabilities.
	The Social Express	The app includes interactive lessons designed to teach children and young adults how to think about and manage social situations and help them to develop meaningful social relationships.
Decision Support (n=7)	AHRQ ePSS	This app was developed to assist primary care clinicians to identify the screening, counseling, and preventive medication services that are appropriate for their patients.
	iDecisio	This app is used to help adults optimize personal decision-making.
	inDecision	This app is designed to simplify choices through the use of pros and cons.
	Informed Consent	This app for researchers is a course that teaches about the key compliance requirements of the informed consent form and the administration process.
	MDS Risk	This app assists physicians with risk stratification and selection of treatment options for patients with Myelodysplastic Syndrome (MDS).
	SecureConsent	This app educates clinical trial candidates and records their informed consent.
	Tools 4 Students	This app includes 25 graphic organizers for students to use to organize their

		thinking while reading or preparing to write.
Clinical Trial (n=3)	Clinical Trial Seek	This app, sourced from clinicaltrials.gov, allows users to get information on cancer trials including how they work and searches by location, disease type, phase of trial and other filters.
	Clinical Trials Mobile	This app, for prospective clinical trial participants or investigators, allows access to the National Library of Medicine database of available clinical trials.
	Guide to Clinical Trails	This app provides clinical trial information and content that is easy for patients to use and understand.
Behavior Modification (n=3)	Behavior Tracker Pro	This app allows teachers, therapists or parents to track behaviors and graph them.
	Skill Tracker Pro	This app helps parents, teachers, therapists, and behavior analysts automate applied behavior analysis instruction for children with autism.
	Ther-Ad for Autism	This evidence- based video self-modeling app includes motivating media, followed by a short clip of the individual with autism engaging in a behavior the parent or teacher wishes to encourage, followed by reinforced motivating content.
